# Advanced Self-Cleaning Surfaces

**DOI:** 10.3390/ma17030537

**Published:** 2024-01-23

**Authors:** Carlo Antonini, Massimiliano D’Arienzo, Michele Ferrari, Maria Vittoria Diamanti

**Affiliations:** 1 Department of Materials Science, University of Milano-Bicocca, 20125 Milan, Italy; carlo.antonini@unimib.it (C.A.); massimiliano.darienzo@unimib.it (M.D.); 2 Institute of Condensed Matter Chemistry and Technologies for Energy, National Research Council, CNR-ICMATE, 16149 Genoa, Italy; michele.ferrari@ge.icmate.cnr.it; 3 Department of Chemistry, Materials and Chemical Engineering “Giulio Natta”, Politecnico di Milano, 20131 Milan, Italy

Hydrophobicity, olephobicity, hemophobicity, amphiphobicity, omniphobicity, icephobicity. In the last fifteen years, an exponentially increasing number of scientific studies have focused on surface micro- and nano-engineering to develop surfaces capable of repelling liquids (and even solids, in the case of ice) by controlling wetting properties. Such interest primarily derives from nature observations, as some plant leaves have developed peculiar wetting properties for self-cleaning purposes, e.g., to remain clean by preventing the adhesion of particles or bacteria on their surfaces [1]. The classic example is Nelumbo nucifera, more commonly known as lotus, but other plants such as Colocasia esculenta, Mutisia decurrens [2] and Salvinia molesta [3] possess similar properties. All these plants are characterized by superhydrophobicity, which combines high water repellence with high mobility: these properties are given by a combination of chemistry (leaves are coated with intrinsically hydrophobic wax) and surface hierarchical topography at the micro- and nano-scale.

Researchers first tried to replicate synthetic superhydrophobic surfaces, but then also extended the concept to all type of liquids (oil, low-surface tension fluids, blood) with an ambitious scientific question: can we design surfaces that can repel any type of liquid, and thus fabricate the perfect self-cleaning surface? The ambition is not a pure academic exercise, but it can have a significant impact on a plethora of applications, spanning from anti-icing to anti-fogging mirrors, low-drag ship hulls, anti-bacterial and anti-viral surfaces, and food containers.

However, the repelling of liquids is not the only strategy to achieve self-cleaning surfaces: the superhydrophilic principle has been adopted to produce self-cleaning glass for building envelopes and photovoltaics, and is already widely diffuse at an industrial level [4]. This effect originates from photoinduced phenomena, and relies on a combined surface hydroxylation and degradation of soiling agents [5].

It is thus clear how self-cleaning surfaces can be achieved by a variety of strategies, materials and technologies, and their applications are as wide as their production methods.

The current interdisciplinary topic, “Advanced Self-Cleaning Surfaces”, has collected a variety of works dedicated to self-cleaning, from theoretical approaches to small-scale laboratory experiments and material validation in relevant environments, demonstrating how tailoring the surface at nano- and micro-meter scale surface phenomena can affect larger scale phenomena. We believe that joining all aspects of such a multifaceted phenomenon into a single collection of articles will help the scientific community involved in this field to improve collaboration among diverse disciplines and promote novel insights across interdisciplinary research fields. On account of its peculiar interdisciplinary character, this article collection was published across different journals: *Materials*, *Membranes*, *Nanomaterials* and *Coatings*. The ten articles included in the topical collection highlight diverse material compositions, production methodologies and applications, as discussed in the following.

Oil–water separation is confirmed to be a hot topic, and has been addressed by several research works with different approaches. A PVDF/DP8/SiO_2_ composite honeycomb membrane was tested for oil separation using five different oil–water mixes, showing high efficiency and good stability and the reusability of the materials developed [6]. Candle soot and polyvinylidene fluoride composites deposited on paper or on aluminium substrates were also evaluated; the materials were considered suitable for such an application due to their superhydrophobicity and superoleophilicity, and their good behaviour was confirmed in tests performed on meshes for both heavy and light oil separation. Such surfaces also proved efficient in protecting stainless steel meshes from concentrated HCl [7]. The specular concept, i.e., developing a superhydrophilic and underwater superoleophobic surface, was applied to obtain oil–water separation with a carbon cloth membrane coated with Cu-doped TiO_2_ and Ag nanoparticles [8]. Water treatment was the objective of another study, based on melt-blending an ethylene–vinyl alcohol copolymer and polypropylene to produce hydrophilic hollow fibres, which were successfully tested for ink separation [9].

In such applications, durability is a key factor to ensure the industrial applicability of such technologies. Several works, indeed, address this issue, including those previously mentioned in which cycles of oil–water separation were run to monitor performance changes. An assessment of surface durability under mechanical abrasion was performed on dendritic electrodeposited copper surfaces, demonstrating how the precursors affect the final quality and durability of the superhydrophobic effect [10].

Durability was also central in studies related to antibacterial, antiviral and antifouling properties of superhydrophobic materials. In this direction, one study reported the production of electrospun polylactide acid nanofiber filters for face masks: polylactic acid was used as the fibre material, and Manka oil was added to impart antiviral, antibacterial and antifungal activity. Durability was assessed by repeated laundering to ensure reusability [11]. An analogous aim was challenged with the development of anti-wetting coatings on PU foams, produced with fluorine-modified silica, able to prevent fouling from bacteria. Material leaching was then evaluated in both polar and non-polar liquids, representative of liquids typically present in both medical and food industries, which are their target application environments: no release was observed in 14 days of immersion, ensuring the good chemical stability of the coating [12]. Bulk modifications, rather than surface treatments, were evaluated instead in a study dedicated to increasing the durability of concrete through the development of a superhydrophobic concrete mix, which was achieved through the addition of isobutyltriethoxysilane (IBTES) and commercial waterproofing agents. Superhydrophobicity in concrete materials is crucial to increase their service life, as most concrete degradation phenomena are water-related [13].

Finally, two studies delved into more general aspects of self-cleaning. On the one hand, the wetting transition mechanism at the basis of superhydrophobicity was addressed in a review work, beginning with the classical theory of static contact angle and then focusing on the effect of surface morphology through the application of fractal theory and re-entrant geometries [14]. On the other hand, more practical aspects were tackled in a research study aimed at understanding the applicability of the ISO 27448:2009 standard for self-cleaning evaluation to current self-cleaning materials, reviewed in 2020 [15]. Indeed, such a standard was developed for smooth, photocatalytic self-cleaning materials, thus making it inapplicable to the increasingly relevant family of superhydrophobic self-cleaning materials [16].

For further information, readers are encouraged to refer to the complete articles.
